# Synthesis, Characterization, and Antibacterial Activities of Novel Sulfonamides Derived through Condensation of Amino Group Containing Drugs, Amino Acids, and Their Analogs

**DOI:** 10.1155/2015/938486

**Published:** 2015-02-24

**Authors:** Muhammad Abdul Qadir, Mahmood Ahmed, Muhammad Iqbal

**Affiliations:** ^1^Institute of Chemistry, University of Punjab, Lahore 54590, Pakistan; ^2^Department of Chemistry, Minhaj University, Lahore, Pakistan

## Abstract

Novel sulfonamides were developed and structures of the new products were confirmed by elemental and spectral analysis (FT-IR, ESI-MS, ^1^HNMR, and ^13^CNMR). In vitro, developed compounds were screened for their antibacterial activities against medically important gram (+) and gram (−) bacterial strains, namely, *S. aureus*, *B. subtilis*, *E. coli*, and *K. pneumoniae*. The antibacterial activities have been determined by measuring MIC values (*μ*g/mL) and zone of inhibitions (mm). Among the tested compounds, it was found that compounds 5a and 9a have most potent activity against *E. coli* with zone of inhibition: 31 ± 0.12 mm (MIC: 7.81 *μ*g/mL) and 30 ± 0.12 mm (MIC: 7.81 *μ*g/mL), respectively, nearly as active as ciprofloxacin (zone of inhibition: 32 ± 0.12 mm). In contrast, all the compounds were totally inactive against the gram (+) *B. subtilis*.

## 1. Introduction

Sulfa drugs containing sulfonamide functional group which have extensive biological activities revolutionised the field of medical sciences [1]. Folic acid, an important chemical for synthesis of bacterial DNA and RNA, is inhibited by sulfonamides; production of new DNA and RNA is decreased by the deficiency of tetrahydrofolate which ultimately decayed the bacteria. Microorganism's normal growth is inhibited due to mistaken attempt by bacteria to convert sulfonamide instead of* p*-amino benzoic acid for synthesis of folic acid. Due to such activities sulfonamides are also efficiently used in agriculture field for antibacterial activities [2, 3]. Inhibition of carbonic anhydrases has been done by the drugs containing sulfonamide functional group and these carbonic anhydrase inhibitors are also reported as potential anticancer, antiglaucoma (as substituted heterocyclic and aromatic sulfonamides), diuretics, and antiobesity agents [4]. Therapeutically sulfonamides are being widely used in human (especially where other antibiotics are nontolerable to patients) and veterinary practice [5]. For agricultural purposes many derivatives of sulfonamide have been reported due to their antifungal [6–8] and herbicidal [9] properties. The newer antibacterial drugs with different mode of action and mechanism have become an emerging demand to overcome existing drugs resistant pathogens. As the pathogenic organisms (bacteria, fungi, and mold) are exposed or treated medically with routine antibiotic drug molecules, they become considerably resistant with emergence of new species as per mutation, conjugation, transduction, or transformation. The synthesis of new sulfonamides has got more attention of researchers for their previous success in the field of pharmaceutical sciences and medicinal chemistry. In recent studies, ten new sulfonamides have been synthesized and characterized by the reaction of p-toluene sulphonyl chloride with essential amino acids (histidine and tryptophan) and amino group containing drugs such as levetiracetam (anticonvulsant), famotidine (antiulcer), celecoxib (NSAID), ribavirin (antiviral), tranexamic acid (antifibrinolytic), furosemide (diuretic), aspartame (nonsaccharide sweetener), and nicotinamide (vitamin B), respectively. Antibacterial activities of synthesized compounds were also evaluated against both gram (+) and gram (−) bacteria.

## 2. Experimental

### 2.1. Chemistry

Chemicals used in present work were of analytical grade obtained from E-Merck (Germany) and BDH (UK) without further purification to synthesize desired compounds; grade 1 quality water (0.01 *μ*S/cm) [10] was prepared in our own laboratory. Alpha IR spectrometer (FTIR-ATR) and Bruker and NMR spectrometer, Bruker, were used to record the IR and ^1^HNMR (500 MHz) and ^13^CNMR (125 MHz) spectra, respectively. PG-T80^+^ UV-vis spectrophotometer and Flash HT Plus elemental analyzer, Thermo Scientific, were used for *λ*_max⁡_ and concentration of hydrogen (H), carbon (C), nitrogen (N), and sulfur (S) of respective synthesized compounds, respectively, while the melting point was measured by Gallenkamp apparatus. JMS-HX-110 spectrometer with electron ionization interface was used for mass spectra. The ^1^HNMR and ^13^CNMR spectra of all the synthesized compounds were measured using MeOD and concentration of all the compounds was 10–20 mg in 0.8–1.0 mL of solvent. Purification and progress of the synthesized compounds were confirmed on precoated TLC silica plate (Merck, Germany).

### 2.2. Antibacterial Activity Assay

*Escherichia coli *ATCC 25922,* Staphylococcus aureus *ATCC 25923*, Bacillus subtilis *ATCC 6633, and* Klebsiella pneumoniae *ATCC 13887 were collected from Mycology Department, University of Punjab, Lahore, Pakistan, and were maintained in tryptic soy agar (TSA) medium slants at 5°C until use. A series of ten 2-fold dilutions were made by dissolving 10–30 mg of each sulfonamide separately in 1 mL dimethyl sulphoxide (DMSO). All the dilutions were made sterile in an autoclave at 121°C for 30 min with 15 psi pressure after filtration through 0.22 *μ*m membrane filter. The minimal inhibitory concentration (MIC) was reported as absence of no observable growth by the lowest concentration of tested compounds after twofold serial dilution. Ten individually numbered test tubes with screw capped are sterilized. Tube 1 was filled with 2 mL of tryptic soy broth culture media including the stock solution of synthesized compounds. 1.0 mL of this solution was introduced into Tube 2 and diluted with 1.0 mL culture media and the procedure was repeated up to Tube 10. The concentration of all the compounds used for MIC value was 2–0.0039 mg/mL obtained by twofold serial dilution technique. The tubes were incubated at 25°C for 72 hrs. Ciprofloxacin was used as reference (positive control to check the sensitivity of tested bacterial strains). 1–3 × 10^8^ cfu/mL of each of gram negative* E. coli *and* K. pneumoniae *and gram positive* S. aureus *and* B. subtilis* were obtained after adjusting the optical density of inoculum at 0.2-0.3 and 0.3-0.4 (620 nm), respectively. All the compounds and reference solutions were applied (50 *μ*L) onto a 6 mm sterile filter paper disc separately and the inoculated plates were incubated at 37°C for 24 hrs. The zones of inhibition (mm) were measured and the antibacterial activities were evaluated. Studies were performed in triplicate and zone of inhibition was calculated with the mean ± SD values.

### 2.3. General Procedure for Synthesis of Sulfonamides

A simple method in aqueous media under dynamic pH control is adopted for synthesis of sulfonamides. Filtration after acidification is involved for isolation of products [11]. All the drugs were weighed accurately and dissolved completely by addition of distilled water by constant stirring using magnetic stirrer. The pH of the reaction contents was strictly monitored and maintained at 8–10 at regular intervals during the experimental reaction using Na_2_CO_3_ solution (1 M). Then p-toluene sulphonyl chloride was accurately weighed and added carefully into the above solution. The reaction was carried in round bottom flask equipped with magnetic stirrer. Alkaline environment made the removal of hydrogen easier. During stirring p-toluene sulphonyl chloride initially floats on the surface and the completion of reaction was examined by the change in pH value due to formation of HCl by the consumption of p-toluene sulphonyl chloride during the reaction. On completion of the reaction pH was adjusted at 2-3 using HCl solution (2 M). The precipitates formed were filtered through Whatman Filter Paper No. 42, washed several times with distilled water and recrystallized using methanol, and finally washed with water and acetone (9 : 1) and dried over anhydrous MgSO_4_. Products formation was confirmed through TLC (methanol : water : acetone in 60 : 20 : 20 ratio).

### 2.4. Spectral Characterization of Sulfonamides

#### 2.4.1. *N*-[(4-Methylphenyl) sulfonyl] Nicotinamide (3a)

611.0 mg (5.0 mmol) nicotinamide reacts with 954.0 mg *p*-toluenesulfonyl chloride (5.0 mmol) and 3a compound was obtained as white solid. IR *υ*_max⁡_ (cm^−1^): 3062 (C-H_aromatic, stretching_), 2978 (C-H_methyl, stretching_), 1705 (C=O_carbonyl_), 1620 (N-H_bending_), 1018 (S=O_stretching_), 1126 (-N- S=O_stretching_), 1458 (C=C-C_aromatic, stretching_), 706 (Ø-S_stretching_); ^1^HNMR (MeOD, *δ*/ppm): 9.11 (s, 1H, CH), 8.98 (d, *J* = 5.08 Hz, 1H, CH), 8.38 (s, 1H, NH), 8.06 (d, *J* = 8.29 Hz, 1H, CH), 7.81 (q, *J* = 7.26 Hz, 1H, CH), 7.42 (d, *J* = 8.21 Hz, 1H, CH), 2.45 (s, 3H, CH_3_); ^13^CNMR (MeOD, *δ*/ppm): 164 (C-7), 151 (C-2), 146.8 (C-6), 137 (C-4), 129 (C-17), 126 (C-18), 22 (C-19); ESI-MS: *m*/*z* 278.48 [M+2]^+^, 276.34 [M]^+^.

#### 2.4.2. (2*S*)-*N*-[(4-Methylphenyl) sulfonyl]-2-(2-oxopyrrolidin-1-yl) Butanamide (3b)

567.0 mg (3.34 mmol) levetiracetam reacts with 635.0 mg *p*-toluenesulfonyl chloride (3.34 mmol) and 3b compound was obtained as white solid. IR *υ*_max⁡_ (cm^−1^): 3363 (N-H_amine, stretching_), 3063 (C-H_aromatic, stretching_), 2960 (C-H_methyl, stretching_), 1720 (C=O_carbonyl_), 1658 (N-H_bending_), 1010 (S=O_stretching_), 1144 (-N- S=O_stretching_), 1504 (C=C-C_aromatic, stretching_), 675 (Ø-S_stretching_); ^1^HNMR (MeOD, *δ*/ppm): 9.68 (br s, 1H, NH), 7.61 (d, *J* = 8.24 Hz, 1H, CH), 7.35 (d, *J* = 8.27 Hz, 1H, CH), 4.43 (t, *J* = 8.86 Hz, H, CH), 3.52 (dt, *J* = 7.21, 1.52 Hz, 2H, CH_2_), 2.45 (s, 3H, CH_3_), 2.41 (dd, *J* = 15.41, 7.42 Hz, 2H, CH_2_), 2.08–2.13 (m, 2H, CH_2_), 0.91 (t, *J* = 7.36 Hz, 3H, CH_3_); ^13^CNMR (MeOD, *δ*/ppm): 172.1 (C-5), 168.3 (C-7), 145.3 (C-19), 135.4 (C-16), 125.9 (C-21), 63.5 (C-6), 44.1 (C-2), 30.8 (C-4), 25.3 (C-10), 17.4 (C-3), 11.9 (C-11); ESI-MS: *m*/*z* 326.25 [M+2]^+^, 324.27 [M]^+^.

#### 2.4.3. 1-[3,4-Dihydroxy-5-(hydroxymethyl)tetrahydrofuran-2-yl]-*N*-[(4-methylphenyl) sulfonyl]-1*H*-1,2, 4-triazole-3-carboxamide (3c)

611.0 mg (2.5 mmol) ribavirin reacts with 477.0 mg *p*-toluenesulfonyl chloride (2.5 mmol) and 3c compound was obtained as white solid. IR *υ*_max⁡_ (cm^−1^): 3371 (N-H_amine, stretching_), 3155 (C-H_aromatic, stretching_), 2962 (C-H_methyl, stretching_), 1711 (C=O_carbonyl_), 1597 (N-H_bending_), 1010 (S=O_stretching_), 1174 (-N- S=O_stretching_), 1496 (C=C-C_aromatic, stretching_), 1357 (O-H_bending_), 678 (Ø-S_stretching_); ^1^HNMR (MeOD, *δ*/ppm): 8.88 (s, 1H, CH), 7.81 (d, *J* = 8.24 Hz, 1H, CH), 7.45 (d, *J* = 8.27 Hz, 1H, CH), 7.03 (br s, H, NH), 5.92 (t, *J* = 5.31, 1H, CH), 4.45 (t, *J* = 5.41, 1H, CH), 4.21 (t, *J* = 5.31, 1H, CH), 4.12 (t, *J* = 5.48, 1H, CH), 3.81 (dd, *J* = 8.8, 5.16 Hz, 2H, CH_2_), 2.43 (s, 3H, CH_3_); ^13^CNMR (MeOD, *δ*/ppm): 157.1 (C-8), 154.1 (C-15), 145.7 (C-24), 140.4 (C-10), 135.4 (C-21), 129.2 (C-23), 91.1 (C-2), 86.8 (C-5), 75.1 (C-3), 69.4 (C-4), 61.9 (C-3), 21 (C-27); ESI-MS: *m*/*z* 400.35 [M+2]^+^, 398.27 [M]^+^.

#### 2.4.4. *N*-[(4-Methylphenyl) sulfonyl] Histidine (5a)

Compound 5a, as white solid, was obtained by the reaction of 518.0 mg (3.34 mmol) histidine with 636.0 mg (3.34 mmol) of *p*-toluenesulfonyl chloride. IR *υ*_max⁡_ (cm^−1^): 3125 (O-H_carboxylic, stretching_), 2962 (C-H_methyl, stretching_), 1591 (N-H_bending_), 1010 (S=O_stretching_), 1170 (-N- S=O_stretching_), 1496 (C=C-C_aromatic, stretching_), 1650 (C=N_stretching_), 678 (Ø-S_stretching_); ^1^HNMR (MeOD, *δ*/ppm): 8.31 (s, 1H, CH), 7.91 (s, 1H, CH), 7.61 (d, *J* = 8.07 Hz, 1H, CH), 7.33 (br s, H, NH), 7.23 (d, *J* = 8.01, 1H, CH), 4.75 (q, *J* = 8.21, 1H, CH), 3.01 (qd, *J* = 14.58, 8.31, 2H, CH_2_), 2.39 (s, 3H, CH_3_); ^13^CNMR (MeOD, *δ*/ppm): 175.1 (C-11), 143.2 (C-17), 135.7 (C-2), 130.1 (C-16), 125.4 (C-15), 53.9 (C-7), 31.2 (C-6), 21.5 (C-21); ESI-MS: *m*/*z* 311.28 [M+2]^+^, 309.29 [M]^+^.

#### 2.4.5. *N*-[(4-Methylphenyl) sulfonyl] Tryptophan (5b)

Compound 5b, as white solid, was obtained by the reaction of 511.0 mg (2.5 mmol) tryptophan with 477.0 mg (2.5 mmol) of *p*-toluenesulfonyl chloride. IR *υ*_max⁡_ (cm^−1^): 3300 (N-H_amine, stretching_), 3025 (O-H_carboxylic, stretching_), 1581 (N-H_bending_), 1040 (S=O_stretching_), 1165 (-N- S=O_stretching_), 1496 (C=C-C_aromatic, stretching_), 708 (Ø-S_stretching_); ^1^HNMR (MeOD, *δ*/ppm): 8.01 (br s, 1H, NH), 7.82 (d, *J* = 7.76 Hz, 1H, CH), 7.71 (s, H, CH), 7.65 (d, *J* = 8.07 Hz, 1H, CH), 7.23 (d, *J* = 8.17 Hz, 1H, CH), 7.13 (t, *J* = 8.11, 1H, CH), 6.75 (d, *J* = 7.71, 1H, CH), 4.61 (t, *J* = 8.31, 1H, CH), 3.50 (dt, *J* = 14.33, 8.23, 2H, CH_2_), 2.39 (s, 3H, CH_3_); ^13^CNMR (MeOD, *δ*/ppm): 175.9 (C-15), 143.5 (C-21), 134.7 (C-2), 130.1 (C-22), 122.9 (C-9), 118.6 (C-4), 111.8 (C-3), 63.5 (C-11), 29.7 (C-10), 21.4 (C-25); ESI-MS: *m*/*z* 360.45 [M+2]^+^, 358.37 [M]^+^.

#### 2.4.6. Methyl *N*-[(4-Methylphenyl) sulfonyl]-*α*-aspartyl-L-phenylalaninate (7a)

White crystals of compound 7a were obtained by charging 589.0 mg aspartame (2.0 mmol) with 381.0 mg of *p*-toluenesulfonyl chloride (2.0 mmol). IR *υ*_max⁡_ (cm^−1^): 3340 (N-H_amine, stretching_), 2950 (O-H_carboxylic, stretching_), 1219 (C-N_amine, stretching_), 1735 (C=O_ester, stretching_) 1581 (N-H_bending_), 1040 (S=O_stretching_), 1170 (-N- S=O_stretching_), 1496 (C=C-C_aromatic, stretching_), 700 (Ø-S_stretching_); ^1^HNMR (MeOD, *δ*/ppm): 7.61 (br s, 1H, NH), 7.42 (d, *J* = 8.06 Hz, 1H, CH), 7.25 (d, *J* = 7.37 Hz, 1H, CH), 6.93 (d, *J* = 8.14 Hz, 1H, CH), 5.33 (d, *J* = 9.11, 1H, CH), 4.65 (d, *J* = 7.67, 1H, CH), 3.61 (s, 3H, CH_3_), 2.93 (dt, *J* = 13.73, 6.83, 2H, CH_2_), 2.39 (s, 3H, CH_3_); ^13^CNMR (MeOD, *δ*/ppm): 174.3 (C-21), 170.7 (C-12), 143.5 (C-27), 136.8 (C-1), 129.7 (C-6), 126.1 (C-4), 55.6 (C-8), 52.3 (C-19), 37.8 (C-7), 21.5 (C-31); ESI-MS: *m*/*z* 450.43 [M+2]^+^, 448.47 [M]^+^.

#### 2.4.7. *trans*-4-({[(4-Methylphenyl)sulfonyl]amino}methyl)cyclohexanecarboxylic Acid (9a)

White crystals of compound 9a were obtained by charging 393.0 mg tranexamic acid (2.5 mmol) with 477.0 mg of* p*-toluenesulfonyl chloride (2.5 mmol). IR *υ*_max⁡_ (cm^−1^): 3263 (N-H_amine, stretching_), 2962 (O-H_carboxylic, stretching_), 1219 (C-N_amine, stretching_), 1597 (N-H_bending_), 1067 (S=O_stretching_), 1157 (-N- S=O_stretching_), 1496 (C=C-C_aromatic, stretching_), 680 (Ø-S_stretching_); ^1^HNMR (MeOD, *δ*/ppm): 7.71 (d, *J* = 8.06 Hz, 1H, CH), 7.56 (br s, H, NH), 7.22 (d, *J* = 8.06 Hz, 1H, CH), 2.85 (d, *J* = 15.17 Hz, 1H, CH), 2.33 (s, 3H, CH_3_), 2.29 (d, *J* = 10.71, 1H, CH), 1.65 (dt, *J* = 11.67, 9.71, 2H, CH_2_), 1.41 (qd, *J* = 12.93, 10.83, 2H, CH_2_); ^13^CNMR (MeOD, *δ*/ppm): 182.5 (C-11), 140.3 (C-17), 136.5 (C-14), 129.5 (C-16), 46.8 (C-7), 38.5 (C-1), 29.6 (C-6), 22.3 (C-21); ESI-MS: *m*/*z* 313.35 [M+2]^+^, 311.37 [M]^+^.

#### 2.4.8. 4-Chloro-2-[(2-furylmethyl)amino]-5-({[(4-methylphenyl)sulfonyl]amino}sulfonyl)benzoic Acid (11a)

Compound 11a, as white solid, was obtained by the reaction of 661.0 mg (2.0 mmol) furosemide with 381.0 mg (2.0 mmol) of *p*-toluenesulfonyl chloride. IR *υ*_max⁡_ (cm^−1^): 3348 (N-H_amine, stretching_), 3250 (O-H_carboxylic, stretching_), 1607 (N-H_bending_), 1072 (S=O_stretching_), 1160 (-N- S=O_stretching_), 1496 (C=C-C_aromatic, stretching_), 702 (Ø-S_stretching_); ^1^HNMR (MeOD, *δ*/ppm): 11.81 (br s, H, NH), 8.42 (s, 1H, CH), 7.89 (d, *J* = 8.17 Hz, 1H, CH), 7.58 (d, *J* = 8.11 Hz, 1H, CH), 7.39 (s, H, CH), 6.65 (s, H, CH), 6.21 (s, H, CH), 6.16 (d, *J* = 3.27, H, CH), 4.41 (s, H, CH), 2.42 (s, H, CH); ^13^CNMR (MeOD, *δ*/ppm): 172.5 (C-14), 153.9 (C-8), 143.5 (C-28), 130.8 (C-27), 123.8 (C-13), 120.5 (C-9), 110.4 (C-4), 42.3 (C-6), 21.5 (C-31); ESI-MS: *m*/*z* 486.95 [M+2]^+^, 484.87 [M]^+^.

#### 2.4.9. 4-Methyl-*N*-({4-[5-(4-methylphenyl)-3-(trifluoromethyl)-1*H*-pyrazol-1-yl]phenyl}sulfonyl) Benzenesulfonamide (11b)

Compound 11b, as white solid, was obtained by the reaction of 424.0 mg (1.11 mmol) celecoxib with 212.0 mg (1.11 mmol) of* p*-toluenesulfonyl chloride. IR *υ*_max⁡_ (cm^−1^): 3327 (N-H_amine, stretching_), 1607 (N-H_bending_), 1065 (S=O_stretching_), 1142 (-N- S=O_stretching_), 1502 (C=C-C_aromatic, stretching_), 1650 (C=N_stretching_), 703 (Ø-S_stretching_), 1095–1125 (C-F_stretching_); ^1^HNMR (MeOD, *δ*/ppm): 16.11 (br s, H, NH), 8.62 (d, *J* = 8.19 Hz, 1H, CH), 8.37 (d, *J* = 8.01 Hz, 1H, CH), 8.08 (d, *J* = 9.91 Hz, 1H, CH), 7.91 (d, *J* = 8.17 Hz, 1H, CH), 7.65 (d, *J* = 7.97 Hz, 1H, CH), 6.81 (s, H, CH), 2.46 (s, H, CH), 2.35 (s, H, CH); ^13^CNMR (MeOD, *δ*/ppm): 147.2 (C-5), 141.8 (C-33), 138.5 (C-13), 131.8 (C-9), 128.3 (C-31), 117.5 (C-12), 110.7 (C-4), 21.5 (C-24); ESI-MS: *m*/*z* 537.55 [M+2]^+^, 535.57 [M]^+^.

#### 2.4.10. (1*E*)-3-[({2-[(Bis {[(4-methylphenyl) sulfonyl] amino} methylene) amino]-1,3-thiazol-4-yl}methyl) thio]-*N*-[(4-methylphenyl) sulfonyl]-*N*′-({[(4-methylphenyl) sulfonyl]amino} sulfonyl) Propanimidamide (13a)

Compound 13a, as white solid, was obtained by the reaction of 282.0 mg famotidine (0.833 mmol) with 635.0 mg of *p*-toluenesulfonyl chloride (3.34 mmol). IR *υ*_max⁡_ (cm^−1^): 3327 (N-H_amine, stretching_), 1674 (N-H_bending_), 1072 (S=O_stretching_), 1172 (-N- S=O_stretching_), 1496 (C=C-C_aromatic, stretching_), 1650 (C=N_stretching_), 702 (Ø-S_stretching_), 652 (C-S_stretching_); ^1^HNMR (MeOD, *δ*/ppm): 9.51 (br s, H, NH), 8.12 (d, *J* = 8.19 Hz, 1H, CH), 7.87 (d, *J* = 8.11 Hz, 1H, CH), 7.68 (d, *J* = 8.91 Hz, 1H, CH), 7.31 (d, *J* = 8.10 Hz, 1H, CH), 3.85 (s, H, CH), 2.93 (d, *J* = 15.97 Hz, 1H, CH), 2.81 (s, 2H, CH_2_), 2.66 (s, 3H, CH_3_), 2.36 (s, 3H, CH_3_), 2.30 (s, 3H, CH_3_); ^13^CNMR (MeOD, *δ*/ppm): 174.8 (C-15), 164.1 (C-2), 153.5 (C-4), 144.4 (C-7), 132.1 (C-54), 126.5 (C-38), 104.5 (C-5), 35.2 (C-11), 32.3 (C-13), 21.2 (C-59); ESI-MS: *m*/*z* 956.21 [M+2]^+^, 954.17 [M]^+^.

## 3. Results and Discussion

**Scheme 1:**
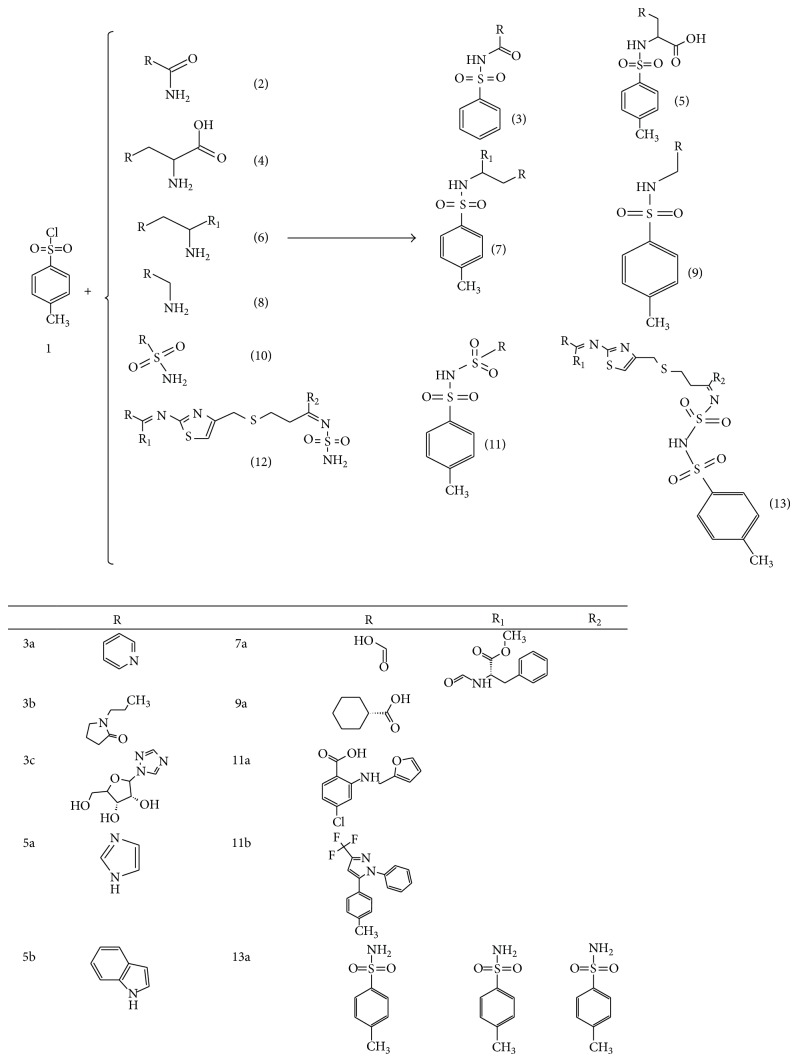
Synthesis of sulfonamides.

A series of ten sulfonamides were synthesized in aqueous basic media by simple reaction of six amino group containing drugs; two amino acids and two amino acid analogs (nicotinamide is used as source of vitamin B and aspartame is sweetener used in various pharmaceutical liquid formulations) with paratoluene sulphonyl chloride with continuous stirring and details of reaction conditions are explained in experimental section and synthetic pathway of sulfonamides is explained in Scheme 1. The compounds 3a and 9a were obtained in excellent yield (above 80%) while the 3c, 5a, and 5b gave the poor yield (below 50%). The remaining compounds were obtained in good yield (69–77%). Elemental analysis was performed for the conformation of all the compounds and measurement of absorption maximum (*λ*_max⁡_) provided the justification. The physiochemical and analytical data of synthesized sulfonamides are presented in Table 1. The synthesized compounds were characterized by FT-IR; the characteristics band at 3263–3371 cm^−1^ of N-H amide stretching and 1174–1127 cm^−1^ for (-N- S=O) and 1072–1010 cm^−1^ (S=O) for all compounds reveals the formation of sulfonamides. [M+2]^+^ peaks obtained by ESI-MS represented the isolation of sulfonyl group in all synthesized compounds. The structures of all the compounds were also confirmed by ^1^HNMR and ^13^CNMR by dissolving in MeOD. ^1^HNMR spectra of compounds 3c, 5a, 7a, and 9a showed a signal at *δ* 7.03–7.61, while a signal at *δ* 16.11 and 11.81 ppm for 11b and 11a corresponds to NH group of sulfonamide.

A broad singlet due to –NH group was also obtained for compounds 3a, 3b, 5b, and 13a at *δ* 8.38, 9.68, 8.01, and 9.51 ppm, respectively. The characteristics C- SO-NH signals at *δ* 131–139 ppm of all the compounds were shown by ^13^CNMR which identified the structures correctly. Synthesized compounds were also screened for their antibacterial activities against gram negative bacterial* E. coli *and* K. pneumoniae *and gram positive* S. aureus *and* B. subtilis* by following the guidelines of CLSI [12, 13] using ciprofloxacin as reference antibacterial agent. Among the bacterial strains, the compounds 3a and 3b have excellent antibacterial activities against* K. pneumoniae *with zone of inhibition comparable with control drug (MIC 62.5). Compounds 3c, 5b, 7a, and 9a showed moderate activities while remaining compounds have no activity against the prescribed bacterial strain. Compounds 5a and 9a exhibited excellent activities against* E. coli *almost the same zone of inhibition as by reference ciprofloxacin (MIC 7.81), while 3a and 7a showed no activity (MIC > 500). The remaining compounds were moderate active against the mentioned strain. All the compounds were totally inactive against the* B. subtilis*. Compounds 3b (MIC 125), 5b (MIC 125), and 11b (MIC 62.5) showed moderate activity, while 11a exhibited poor activity against the* S. aureus* and remaining compounds are totally inactive. The MIC values and zone of inhibitions are presented in Table 2.

## 4. Conclusion

In conclusion, ten novel sulfonamides were synthesized; the reactions conditions are easy and excellent yields of compounds were obtained and progress of reaction was monitored by TLC and their structures were confirmed by spectral and elemental analysis. All the synthesized compounds were evaluated for their antibacterial activities and the results of their bioassay indicated that the sulfonamides attached to amino acid (histidine) and antifibrinolytic (tranexamic acid) showed antibacterial activities comparable to ciprofloxacin although these two agents alone have no antibacterial activity. The results confirmed that the compounds which are inactive against bacterial strains showed antibacterial activities after formation of sulfonamides.

## Figures and Tables

**Table 1:** Physiochemical and analytical data of sulfonamides.

Compounds	M.P. (°C)	Time (h)	Yield (%)	*R*_*f*_^a^ value	*λ*_max⁡_^b^ (nm)	Molecular formula (Mol. Wt.)	Elemental analysis (found/cal.) %			
							C	H	N	S
3a	178–180	3	84.8	0.72	219	C_13_H_12_N_2_O_3_S (276.31)	56.53/56.51	4.35/4.38	10.12/10.16	11.63/11.60
3b	270–272	3	76.5	0.61	221	C_15_H_20_N_2_O_4_S (324.40)	55.50/55.54	6.19/6.21	8.68/8.64	9.82/9.88
3c	174–176	3	47.3	0.57	229	C_15_H_18_N_4_O_7_S (398.40)	45.13/45.22	4.58/4.55	14.10/14.06	8.01/8.05
5a	184–186	3	33.1	0.53	223	C_13_H_15_N_3_O_4_S (309.34)	50.43/50.47	4.85/4.89	13.52/13.58	10.33/10.37
5b	136–138	3	41.5	0.67	277	C_18_H_18_N_2_O_4_S (358.41)	60.28/60.32	5.1/5.06	7.88/7.82	8.92/8.95
7a	207–209	3	69.8	0.61	257	C_21_H_24_N_2_O_7_S (448.49)	56.13/56.24	5.28/5.39	6.26/6.25	7.11/7.15
9a	145–147	3	81.9	0.72	229	C_15_H_21_NO_4_S (311.40)	57.83/57.86	6.75/6.80	4.42/4.50	10.23/10.30
11a	186–188	3	77.5	0.68	233	C_19_H_17_N_2_O_7_S_2_Cl (484.93)	47.10/47.06	3.46/3.53	5.81/5.78	13.15/13.22
11b	148–150	3	76.9	0.72	235	C_24_H_20_F_3_N_3_O_4_S_2_ (535.56)	53.73/53.82	3.72/3.76	7.76/7.85	11.86/11.97
13a	95–97	3	76.7	0.76	274	C_36_H_39_N_7_O_10_S_7_ (954.20)	45.33/45.31	4.05/4.12	10.19/10.28	23.46/23.52

^a^ *R*_*f*_ value measured in methanol : water : acetone in 60 : 20 : 20 ratio.

^b^ *λ*_max⁡_ measured in methanol.

**Table 2:** Zone^a^ of inhibition and MIC^b^ of sulfonamides against pathogenic bacterial strains.

Compounds	Name of bacteria							
	Gram (+) bacterial strains				Gram (−) bacterial strains			
	*S. aureus* (ATCC 25923)		*B. subtilis* (ATCC 6633)		*E. coli* (ATCC 25922)		*K. pneumoniae* (ATCC 13887)	
	Zone of inhibition	MIC	Zone of inhibition	MIC	Zone of inhibition	MIC	Zone of inhibition	MIC
3a	—	>500	—	>500	—	>500	26 ± 1.33	62.5
3b	19 ± 1.22	125	—	>500	28 ± 0.91	15.63	28 ± 0.33	62.5
3c	—	>500	—	>500	18 ± 1.32	125	—	>500
5a	—	>500	—	>500	31 ± 0.12	7.81	18 ± 0.38	125
5b	19 ± 0.81	125	—	>500	18 ± 0.48	125	—	>500
7a	—	>500	—	>500	—	>500	—	>500
9a	—	>500	—	>500	30 ± 0.12	7.81	—	>500
11a	12 ± 0.92	250	—	>500	18 ± 1.51	250	16 ± 0.55	250
11b	18 ± 1.04	62.5	—	>500	20 ± 1.90	250	18 ± 0.87	250
13a	—	>500	—	>500	28 ± 0.53	125	18 ± 0.91	125
Ciprofloxacin^c^	32 ± 0.22	1.25	32 ± 0.13	1.25	32 ± 0.12	0.625	30 ± 0.12	0.625

^a^Zone of inhibition was measured in mm.

^b^MIC (minimum inhibitory concentrations) were measured in *μ*g/mL.

^c^Control drug.
